# Time to Reconsidering the Potential Role of Leech Salivary Proteins in Medicine: Type-II Kounis Syndrome Triggered by Leech Bite

**DOI:** 10.12669/pjms.41.1.10565

**Published:** 2025-01

**Authors:** Sedat Tas, Bekir Serhat Yildiz, Alihan Ersin, Ummu Tas

**Affiliations:** 1 Sedat Tas, Associate Professor, Department of Cardiology, Manisa Celal Bayar University, Yunusemre, Manisa, Turkey; 2 Bekir Serhat Yildiz, Associate Professor, Department of Cardiology, Manisa Celal Bayar University, Yunusemre, Manisa, Turkey; 3 Alihan Ersin, Associate Professor, Department of Cardiology, Manisa Celal Bayar University, Yunusemre, Manisa, Turkey; 4 Ummu Tas, Associate Professor, Department of Cardiology, Izmir Demokrasi University, Goztepe, Izmir, Turkey

**Keywords:** Leech, Kounis syndrome, Anaflaxia, Myocardial infarction

## Abstract

Kounis syndrome also known as allergic myocardial infarction, represents the simultaneous occurrence of acute coronary syndromes with allergic or hypersensitivity reactions. We present a case of a 58-years-old male who developed anaphylaxis following a leech bite, leading to myocardial infarction despite the absence of prior allergic history. He was entubated and cardiopulmonary resusciation had been performed for 10 minutes. The patient was successfully resuscitated and Intravenous antihistamine, prednisolone and adrenaline were given. The patient was stabilized and transferred to the intensive care unit and coronary angiography was performed, which revealed a 90% stenosis in the circumflex coronary artery. A stent was successfully implanted in the affected artery. This report emphasizes the complexity of diagnosing and managing Type-II Kounis syndrome and highlights the need for increased clinical awareness.

## INTRODUCTION

Kounis syndrome is a rare but increasingly recognized condition characterized by the coexistence of ACS with allergic or hypersensitivity reactions.^1^ It involves inflammatory mediators that precipitate coronary artery spasm or atheromatous plaque rupture. There are three types of Kounis syndrome.^2^ Type-II Kounis syndrome, specifically known as allergic myocardial infarction, is characterized by ACS triggered by allergic reactions.^3^

In Type-II Kounis syndrome, patients with pre-existing atherosclerosis may experience an inflammatory response that destabilizes atherosclerotic plaques.^4^ The inflammatory mediators increase the expression of adhesion molecules and attract more inflammatory cells to the site of the plaque. This can lead to plaque rupture, exposing the thrombogenic core to the bloodstream, and resulting in thrombus formation. Various allergens, including drugs, foods, insect stings, or environmental factors can trigger this process. Leeches are among those allergens.

Although they are used for therapeutic purposes against various diseases, especially due to their anticoagulant and anti-inflammatory effect, their saliva contains allergenic proteins that can cause anaphylaxis and even death. Therefore, understanding the clinical presentation and management strategies is crucial for improving patient outcomes. This case report details the presentation, diagnostic process, and management of a patient with Type-II Kounis syndrome triggered by a leech bite. Our case is the first in the literatüre in terms of Type-II Kounis syndrome caused by a leech bite. Informed consent was obtained from the patient included in the study.

## CASE PRESENTATION

A 58-years-old male with no known history of allergies or comorbidities was admitted to the emergency department with signs of anaphylaxis. According to his wife, the patient experienced sudden onset of facial edema, shortness of breath, and chest pain following a leech bite on the medial side of his right lower leg. He was entubated and cardiopulmonary resuscitation had been performed for 10 minutes by first aid team. Upon arrival to the emergency room, the patient was in severe distress, requiring intubation and mechanical ventilation. Cardiopulmonary resuscitation had been ongoing for 10 minutes, during which the patient was defibrillated four times due to recurrent ventricular fibrillation. The patient was successfully resuscitated. Intravenous antihistamine, prednisolone and adrenaline were given. Blood pressure was maintained at 120/70 mmHg with the aid of a noradrenaline infusion, and his pulse rate was 126 beats per by minute.

The patient was stabilized and transferred to the intensive care unit. The patient was extubated and Noradrenaline was ended. An electrocardiogram (ECG) showed ST segment depression in v2-v5 leads at the time of admission and ST segment depression improved at discharge. The rhythm was atrial fibrillation. Furthermore, serum high sensitive troponin levels were significantly elevated to 22.000 ng/l, indicating myocardial injury (Table-I). A bedside echocardiogram revealed normal cardiac function.

**Table-I:** The laboratory findings of the patient.

hS-Troponin (ng/l)	22.000	WBC (x109/L)	12.9
Glu (mg/dl)	174	NEU (x109/L)	11.3
Cre (mg/dl)	1.18	LYM (x109/L)	1.0
AST (U/L)	79	MONO (x109/L)	0.5
ALT (U/L)	63	EOS (x109/L)	0.01
Alb (mg/dl)	4.3	RBC (x109/L)	5.2
Na (mmol/l)	139	HGB (g/dl)	16.3
K (mmol/l)	4.2	HCT (%)	49.8
Cl (mmol/l)	92	MCV (fL)	95.8
Ca (mmol/l)	9.3	PLT (x109/L)	166
Mg (mmol/l)	2.4	RDW (%)	13
CRP	0.7	MPV (fL)	10.2
Uric acid	7.6	PDW (%)	15.9
		PCT (%)	0.17
		PT (sc)	11.3
		PTT (%)	102
		PT INR	0.96
		APTT	28.4

Alb: Albumin, ALT: Alanin aminotransferase, APTT: Active Partial thromboplastin time, AST: Aspartate aminotransferase, Ca: Calcium, Cl: Chlor, Cre: Creatinin, EOS: Eosinophil, Glu: Glukose, HB: Hemoglobin, HCT: Hematocrit, K: Potassium, LYM: Lymphocyte, MCV: Mean corpuscular volüme, MPV: Mean platelet volüme, Mg: Magnesium, MONO: Monocyte, Na: Sodium, PCT:Plateletcrit, PDW: Platelet distribution width, PLT: Platelet, PNL: polymorphonuclear leukocytes, PT: Prothrombin time, PT INR: Prothrombin time international normalized ratio, PTT: Partial thromboplastin time, RBC:Red blood cell, RDW: Red cell distribution width, WBC: White blood cell.

**Fig.1:**
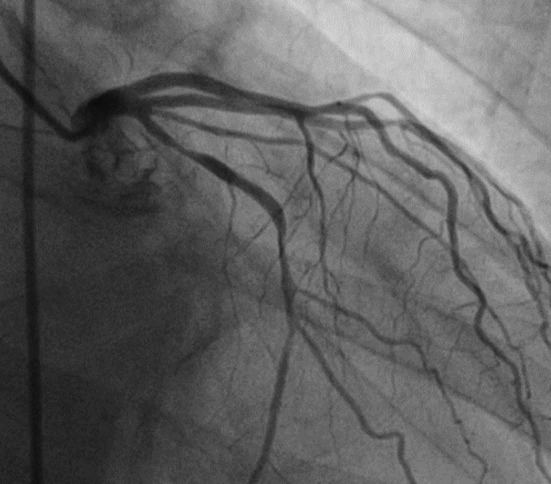
The coronary angiogram, shown here in the left caudal view, appears to demonstrate a significant lesion in the circumflex coronary artery.

**Fig.2:**
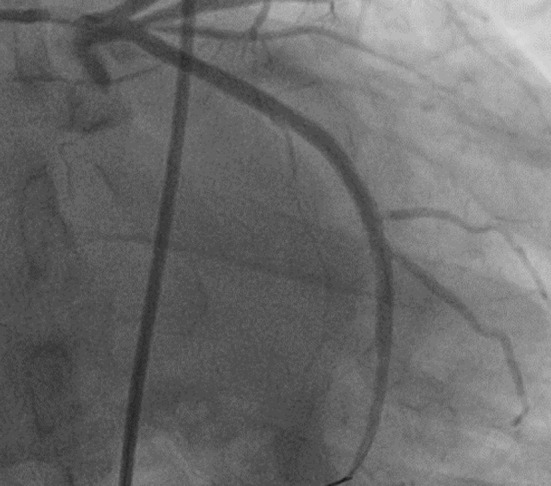
The coronary angiogram, shown here in the left caudal view, appears to demonstrate final view of the circumflex coronary artery.

Given the clinical presentation and elevated troponin levels, coronary angiography was performed, which revealed a 90% stenosis in the circumflex coronary artery (Fig.1). A stent was successfully implanted in the affected artery (Fig.2). By this way the patient diagnosed as Type-II Kounis Syndrome and the patient was placed on an antiplatelet therapy regimen.

## DISCUSSION

Kounis syndrome can be categorized into three types: Type-I occurs in patients with normal coronary arteries and no predisposing factors for coronary artery disease,^5^ Type-II occurs in patients with pre-existing atherosclerotic disease; and Type-III in patients with stent thrombosis. This case aligns with Type-II Kounis syndrome, where an acute allergic reaction precipitates myocardial infarction due to pre-existing coronary artery disease.

The pathophysiology of Kounis syndrome involves the activation of mast cells and the release of inflammatory mediators such as histamine, leukotrienes, and cytokines during an allergic reaction.^6^ These mediators can induce coronary vasospasm and, in patients with underlying atherosclerotic disease, can lead to plaque rupture and thrombosis. In our case, the responsible factor was interstingly a leech, which has cardio protective effects by the help of anticoagulant and anti-inflammatory effects and is used as a medical treatment option against various diseases.^7^

This case is notable as the trigger was a leech bite, a rare cause of anaphylaxis leading to Kounis syndrome. The elevated troponin levels despite absence of significant ECG changes, highlights the importance of considering Kounis syndrome in differential diagnosis when patients present with simultaneous anaphylaxis and chest pain.

The management of Kounis syndrome involves addressing both the allergic reaction and the myocardial ischemia.^8^ In this case, initial treatment included intubation, mechanical ventilation, and steroid administration. Significant coronary artery stenosis was identified, requiring percutaneous coronary intervention with stent placement.

Immediate management focused on stabilizing the patient’s cardiovascular and respiratory status. Intubation and mechanical ventilation were crucial due to severe anaphylaxis, while steroid infusion aided in managing the allergic reaction. Given the myocardial ischemia indicated with elevated troponin levels, coronary angiography was performed, revealing a significant stenosis in the circumflex coronary artery, which was successfully stented. The patient was initiated on dual antiplatelet therapy with aspirin and clopidogrel to prevent the risk of stent thrombosis. Further management included monitoring for potential recurrence of anaphylaxis and myocardial ischemia. The patient was advised to avoid potential allergens and referred to an allergist for further evaluation and potential desensitization therapy.

## CONCLUSION

This case highlights the diagnostic and therapeutic challenges of Type-II Kounis syndrome, particularly in the context of unusual allergic triggers such as a leech bite. Timely identification and management of both the allergic reaction and myocardial ischemia are pivotal in enhancing patient outcomes. Increased awareness and further research into the mechanisms and management of Type-II Kounis syndrome are essential for advancing clinical practice.

### Author’s contributions:

**ST:** Conceptualization, methodology, investigation, and writing.

**BSY:** Data curation, validation, visualization, and writing.

**UT:** Literature search, Review and editing.

**AE:** Literature search, Investigation, and writing.

All authors reviewed the results and approved the final version of the manuscript.
